# Automatic continuous P_0.1_ measurements during weaning from mechanical ventilation: a clinical study

**DOI:** 10.1186/s13613-025-01455-x

**Published:** 2025-04-01

**Authors:** Flora Delamaire, Maamar Adel, Guillot Pauline, Quelven Quentin, Coirier Valentin, Painvin Benoit, Tadie Jean-Marc, Terzi Nicolas, Gacouin Arnaud

**Affiliations:** 1 Maladies Infectieuses et Réanimation Médicale, CHU de Rennes, Rennes, F-35033 France; 2 Université de Rennes 1, Faculté de Médecine, Rennes, F-35043 France; 3 Inserm-CIC-1414, Faculté de Médecine, Université de Rennes 1, IFR 140, Rennes, F-35033 France

**Keywords:** P_0.1_ automatic measurement, Mechanical ventilation weaning, Ventilator accuracy, Spontaneous breathing trial

## Abstract

**Background:**

In critically ill patients, weaning from mechanical ventilation (MV) includes spontaneous breathing trial (SBT) usually followed by a reventilation period in order to recover from the alveolar derecruitement induced by the SBT. The measurement of occlusion pressure during the first 100 ms of an airway occlusion (P_0.1_) one of the non-invasive tools available for estimating the respiratory drive, is a determinant of patient respiratory effort. This clinical study explores the use of non-invasive continuous monitoring of occlusion pressure automatically calculated by ventilators in the first 100 ms of airway occlusion (P_0.1_ *vent*) during SBT and reventilation periods. The study aimed to investigate patient or respirator factors influencing P_0.1 *vent*_ as well as the association of P_0.1 *vent*_ values with extubation success or failure.

**Patients and Methods:**

This prospective observational study, conducted from February 2022 to April 2023, included adult patients intubated for more than 24 h and screened for extubation weaning. SBTs were performed for one hour with zero pressure support and zero end-expiratory pressure (PS0 ZEEP). Reventilation followed for an hour with pressure support (8–12 cmH_2_O) and PEEP (5 cmH_2_O). Data included patient characteristics, ventilator parameters and extubation outcomes.

**Results:**

The study involved 224 measurements from 212 patients, with 157 successful extubations, 46 extubation failures at day 7 and 21 SBT failures. P_0.1 *vent*_ mean values were significantly higher for extubation failures and SBT failures compared to successful extubations (*p* < 0.001). Delta P_0.1 *vent*_ ((P_0.1 *vent*_ reventilation - P_0.1 *vent*_ SBT)/ P_0.1 *vent*_ SBT) was significantly different according to whether extubation was a success or a failure: 0.21 (0.02–0.62) cm H_2_O vs. P_0.1 *vent*_ vs. 1.12 (0.54–2.38) cm H_2_O; *p* < 0.0001 respectively. Values significantly differed in both the SBT and the reventilation periods whether or not patients had previous ARDS: 1.08 (0.70; 2.02) cmH_2_O vs. 0.80 (0.54; 1.28) cmH_2_O respectively (*p* = 0.003). Noteworthy, P_0.1 *vent*_ values were influenced by airway humidification systems (0.92 (0.57; 1.54) cmH_2_O with humidification vs. 1.27 (0.91; 2.24) cmH_2_O without, *p* = 0.003).

**Conclusion:**

The delta of P_0.1_*vent* values between SBT and reventilation are higher for patients who fail extubation, especially for those who had ARDS. While elevated P_0.1 *vent*_ values were associated with extubation failure, the overlap in values limits its usefulness as a reliable predictor.

**Supplementary Information:**

The online version contains supplementary material available at 10.1186/s13613-025-01455-x.

## Background

In the intensive care unit (ICU) setting, weaning from mechanical ventilation (MV) is a challenging period requiring the most appropriate assessment of the patient’s ability to breathe without using a ventilator. The spontaneous breathing trial (SBT) aims to estimate the patients’ respiratory drive, usually defined as the intensity of the output of respiratory centers which determines the effort exerted in each breath [1]. SBT can be performed in pressure support (PS) or with a T-piece [2], and is usually followed by a period of reventilation. Whether reconnection to the ventilator for 1 h following successful SBT may prevent reintubation is debated [3, 4], a recent study has shown the value of this reventilation period in recovering from the alveolar derecruitment induced by the SBT [5]. The physiological mechanisms of this derecruitment remain unclear, but assessment of the patient’s respiratory drive and neural breathing during this period seems crucial. One of the non-invasive tools available for estimating the respiratory drive is the measurement of drop-in airway pressure 100 milliseconds after the onset of inspiration during an end-expiratory occlusion of the airway(P_0.1_) [6–9]. P_0.1_ measurement is available on most ventilators but requires an occlusion maneuver. However, recently automated estimation without occlusion has been developed and allows non-invasive continuous monitoring of P_0.1_, usually named P_0.1_*vent* [6, 10]. Some studies suggest that P_0.1_*vent* is a reliable assessment of P_0.1_ [7–10]. However, data on P_0.1_*vent* are mainly bench-test data and few clinical data are available [7–12]. To the best of our knowledge, P_0.1_*vent* has been very few assessed in real-life study. Since P_0.1_*vent* gives an estimate of respiratory drive, monitoring it is of interest at the time of weaning from MV in order to assess its evolution during the spontaneous breathing trial (SBT) and the reventilation period [3, 5]. We hypothesized that P_0.1_ during the SBT and the reventilation period differs between successful and unsuccessful extubations. For that purpose, we conducted a prospective observational pilot study to monitor and evaluate variations in P_0.1_*vent* during the SBT and reventilation period from February 2022 to April 2023.

## Patients and methods

### Study population

The study was approved by the Ethics Committee of the Centre Hospitalier Universitaire de Rennes (Ethics committee approval number 22.216). All adult (≥ 18 years) patients intubated for more than 24 h and mechanically ventilated using a Servo U ventilator (Getinge group, Servo-u^®^, Solna, Sweden), without do not reintubate order and screened for MV weaning were included. Patients were identified ready to undergo an SBT using criteria from the international conference consensus on weaning [13, 14]; the main criteria for SBT were a response to simple orders with hemodynamic stability and ventilatory criteria with FiO2 ≤ 50% and PEEP < 10 cmH_2_O (See supplemental Table S1).

### Ventilation equipment

Servo U (Getinge group, Servo-u^®^, Solna, Sweden) ventilators were used during the study. Servo U ventilators estimate P_0.1_*vent* calculating the steepest tangent of the drop-in pressure curve during an inspiratory effort without occlusion maneuver. Airway humidification system was notified: filter or heated humidifier. The ventilator tubing was identical for all patients (length: 1.6 m, intersurgical Ltd, UK) and the inspiratory trigger was set at 1 l/min for all patients. We excluded ventilators that measure P_0.1_ with occlusion maneuver. Hamilton C6 ventilators that also estimate P_0.1_ were excluded, as, to the best of our knowledge, the P_0.1 *vent*_ provided by the Hamilton C6 could be underestimated [15, 16].

### Study procedure

In our 30-bed ICU of a tertiary teaching hospital, SBT duration is planned for 1 h in zero pressure support with zero end expiratory pressure (PS0 ZEEP) [17] in all subjects and stopped earlier when poorly tolerated. After the SBT, patients are reventilated in pressure support between 8 and 12 cmH_2_O and 5 cmH_2_O of end expiratory pressure during 1 h before extubation [3, 5]. During these two hours (1 h of PS0 ZEEP plus one hour of reventilation in PS mode), respiratory and heart rates, pulse oximetry (SpO_2_), arterial blood pressure, transcutaneous PCO_2_ (PtcCO_2_) (TCM5, Radiometer, Copenhagen, Denmark) [18] and P_0.1_*vent* were monitored and recorded at 0, 15, 30, 45, and 60 min after the start of the SBT and 0, 15, 30, 45, and 60 min after the end of the SBT defined as the reventilation period before extubation. If the SBT was stopped earlier for poor tolerance reventilation data were recorded as well. In accordance with guidelines and previous studies, the following criteria were used in our weaning protocol to define SBT failure: development during the SBT of any of the following events including respiratory rate (RR) > 35 breaths/min, increased accessory muscle activity, SpO_2_ persistently less than 90% despite increasing FiO_2_, heart rate persistently greater than 140 beats/min, systolic blood pressure < 90 or > 180 mmHg, appearance of cyanosis or mottling, depressed mental status or agitation [13, 14, 19]. Extubations were performed with the assistance of a nurse and a physiotherapist under the physician’s control. Patients were managed after extubation according to the HIGH-WEAN study criteria for post-extubation prophylactic NIV administration [20]. NIV was carried out in PS mode with a minimal PS level of 5 cm H_2_O targeting a tidal volume around 6 to 8 mL/kg of predicted body weight, a PEEP level between 5 and 10 cm H_2_O, and FiO_2_ adjusted to obtain adequate oxygenation (SpO_2_ ≥ 92%). Extubation failures were defined as the need for reintubation within 7 days after extubation [19]. We also included 6 postoperative liver transplant recipients without respiratory or hemodynamic failure and ventilated less than 24 h for whom the physician in charge decided a SBT. These patients served as controls.

### Data collection

**Fig. 1:**
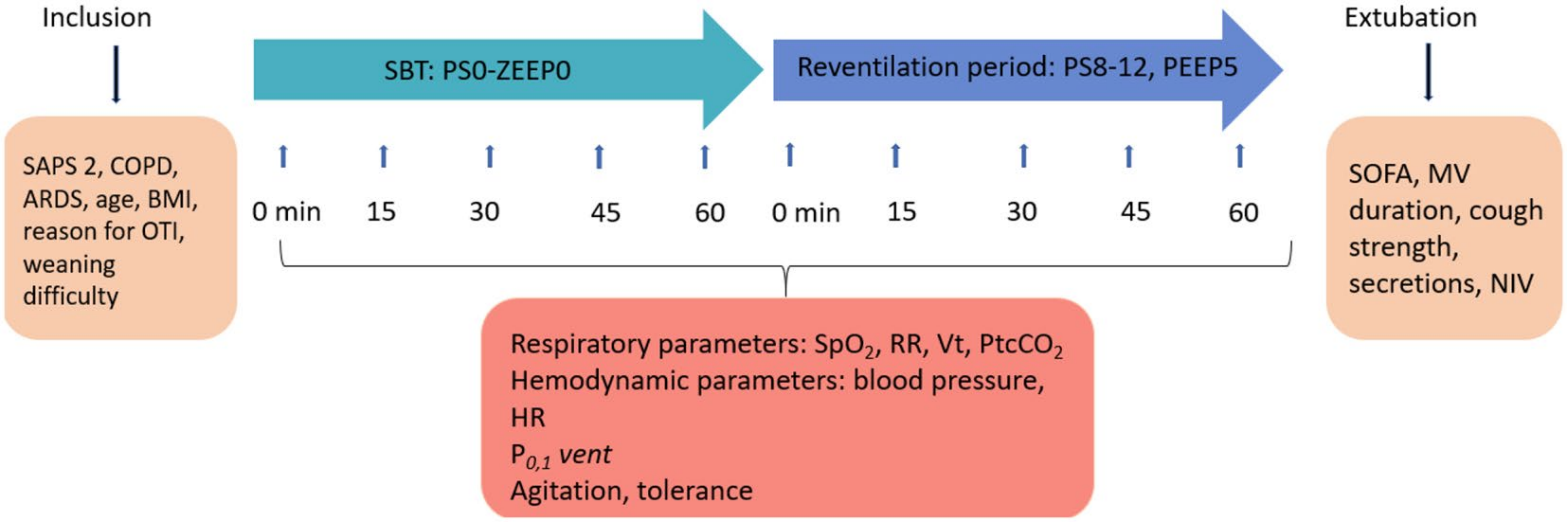
Data collection

For the purposes of this study, a session was defined as the period of SBT and the following reventilation period. In addition to the variables monitored during SBT and the reventilation period, the following data were prospectively recorded: baseline characteristics of subjects at admission, including simplified acute physiology score (SAPS II) [21], diagnosis of moderate to severe acute respiratory distress syndrome (ARDS) [22], SARS-CoV-2 pulmonary infection, risk factors for extubation failure (chronic cardiac or respiratory disease, age > 65 y [20]), body mass index, the main reason for intubation and use of a heated humidification. Furthermore, based on the European consensus [19], patients were classified for weaning difficulty independently of extubation success or failure and based exclusively on the fact that the patient was extubated or not: extubated within 24 h after the first SBT defined as simple weaning, extubated more than 24 h and fewer than 7 days after the first unsuccessful SBT defined as difficult weaning, and extubated more than 7 days after the first unsuccessful SBT defined as prolonged weaning. At the time of extubation SBT, the following data were recorded: sequential organ failure assessment (SOFA) scores [23], last blood gas value, previous duration of MV, number of previous SBTs without extubation, ventilator settings, quality of cough strength and amount of secretions assessed by nurses (no secretion, few, abundant, very abundant). Variables recorded after extubation included the following: treatment with noninvasive ventilation, time and reason for reintubation, length of ICU stay, and ICU and hospital mortality (Fig. 1). The ventilatory ratio was estimated with PtcO_2_ with the formula: (RR x tidal volume x PtcCO_2_)/(ideal body weight x 100 x temperature) [24, 25]. All subjects in the study were assessed for the first extubation during the ICU stay. Extubated patients were studied only once.

### Statistical analysis

The statistical analysis was carried out in two steps. The first stage assessed the factors that could influence the P_0.1_*vent* results. In a second step, data and especially P_0.1_*vent* results were compared between patients who succeeded and patients who failed extubation at day 7. We performed an exploratory analysis; consequently, there was no definitive way for patient sample size to be calculated. We considered that a minimum of 30 patients who failed extubation was required to perform parametric tests. However, continuous variables appeared non-normally distributed when using the Shapiro-Wilk test. Consequently, continuous variables are described as medians and interquartile range (IQR). P_0.1_*vent* recorded at the five time points were pooled during the SBT and the reventilation period. The Wilcoxon rank-sum test was used for comparisons of continuous data between two groups; the Kruskal Wallis test was used for comparisons between three groups or more. Regarding the analysis of repeated values, it was not possible to use ANOVA after assessment of normality and sphericity. Consequently, we used a linear mixed model for repeated values. Variables were included as fixed effect and we used a random intercept to take into account heterogeneity across subjects.

We performed a logistic regression analysis to determine whether the P_01 *vent*_ recorded during the SBT period and/or during the ventilation period were independently associated with failure of extubation on day 7 after adjustment for ARDS and heated humidifier. Results are expressed as odds ratios (OR) with 95% confidence intervals (CI). To assess the reproducibility of the model for the recorded P_01 *vent*_ independently associated with extubation failure, we implemented a cross-validation procedure. The dataset was randomly split into training (70%) and test (30%) subsets, ensuring reproducibility with a fixed random seed. The model was trained on the training subset and validated on the test subset. Predictions were made using a 0.5 threshold for classification. Performance was assessed through the confusion matrix, accuracy, and the Area Under the Receiving Operating Characteristic (ROC) curve (AUC) (supplemental Fig. S2). In addition, variability of P_0.1_*vent* and respiratory rate were assessed by the coefficient of variation (standard deviation divided by the mean; the higher the coefficient of variation, the higher the variability). Proportions are described as percentages and the Chi squared test or Fisher exact test were used as required for comparisons. The test for correlation used was the Spearman correlation test. All statistical analyses were performed using R 4.2.2 (R Foundation for Statistical Computing, Vienna, Austria). P values < 0.05 were considered statistically significant.

## Results

### Patients

**Fig. 2:**
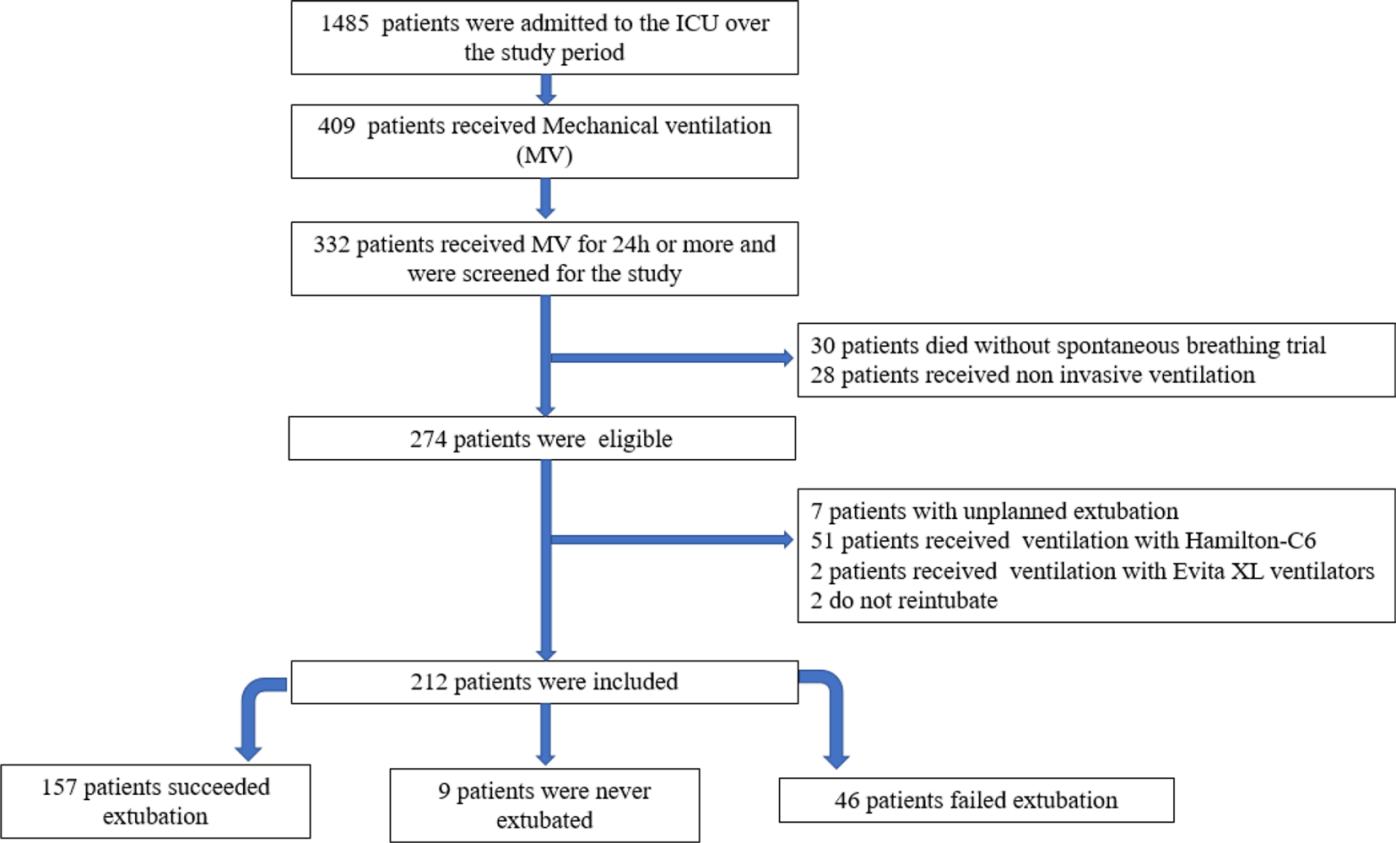
Flow chart of patients

From February 11 2022, to April 8 2023, 1485 patients were admitted to our ICU, 409 received MV, among whom 332 received MV for more than 24 h. Thirty died before the first SBT and 28 received non-invasive mechanical ventilation. Two hundred and seventy-four patients were screened for the study and 212 were included: Fig. 2 Flow chart.

Patient characteristics of those who failed extubation and those who succeeded extubation were similar, notably the proportion of patients with COPD, ARDS and obesity (Table 1). The SAPS II score did not significantly differ between the two groups of patients. On the day of extubation, prior duration of MV was significantly longer in the extubation failure group than in the success group, the SOFA score was higher, and cough was less efficient.

**Table 1:** Characteristics compared between patients who succeeded and patients who failed extubation

Variables	All extubated patients		Issue of extubation at day 7		*P* value
	*N* = 203		Failure *N* = 46	Success *N* = 157	
Age, years	63 (51,69)		63 (55,68)	62 (50,69)	0.49
Male gender	135 (67)		33 (72)	102 (65)	0.50
SAPS II, points	55 (43,70)		60 (44,74)	55 (43,70)	0.26
**Comorbidities**					
Obesity, n (%)	71 (35)		16 (35)	55 (35)	1
Cardiac disease, n (%)	41 (20)		8 (17)	33 (21)	0.74
Neurological disease, n (%)	43 (23)		16 (35)	30 (19)	**0.04**
COPD, n (%)	37 (17)		10 (22)	24 (15)	0.42
OHS, n (%)	23 (11)		6 (13)	17 (11)	0.88
ARDS, n (%)	54 (27)		14 (30)	40 (26)	0.63
Covid19, n (%)	18 (9)		4 (9)	14 (9)	1
**Data the day of extubation**					
Previous length of MV, days	9 (4,16)		10 (5,15)	9 (4,16)	0.30
SOFA score, points	3 (2,4)		3 (2,4)	2 (2,3)	**0.002**
Ability to raise arms, n (%)	63 (11)		16 (35)	47 (30)	0.66
Ineffective cough, n (%)	74 (37)		24 (52)	50 (32)	**0.02**
Abundant secretions, n (%)	52 (25)		18 (39)	34 (22)	**0.004**
Pressure support ventilation, n (%)	169 (83)		36 (78)	133 (85)	0.37
**Last blood gas values before extubation**					
PaO_2_, mmHg	81 (70,95)		79 (70,94)	82 (70,94)	0.64
PaCO_2_, mmHg	38 (35,42)		39 (36,42)	37 (34,41)	**0.03**
PH	7.44 (7.39,7.48)		7.44 (7.39,7.48)	7.40 (7.44,7.48)	0.92
**After extubation**					
Prophylactic NIV, n (%)	123 (61)		30 (65)	93 (59)	0.56
Weaning difficulty, n (%) Simple Difficult Prolonged	152 (75) 31 (15) 20 (10)		16 (35) 16 (35) 14 (30)	136 (86) 15 (10) 6 (4)	**< 0.001**
Alive at ICU discharge, n (%)	199 (98)		34 (96)	155 (98)	0.45

Continuous variables are expressed by medians (interquartile range) and quantitative data by numbers (percentage)

### Comparison of P_0.1_ vent during sessions with different outcomes

**Fig. 3:**
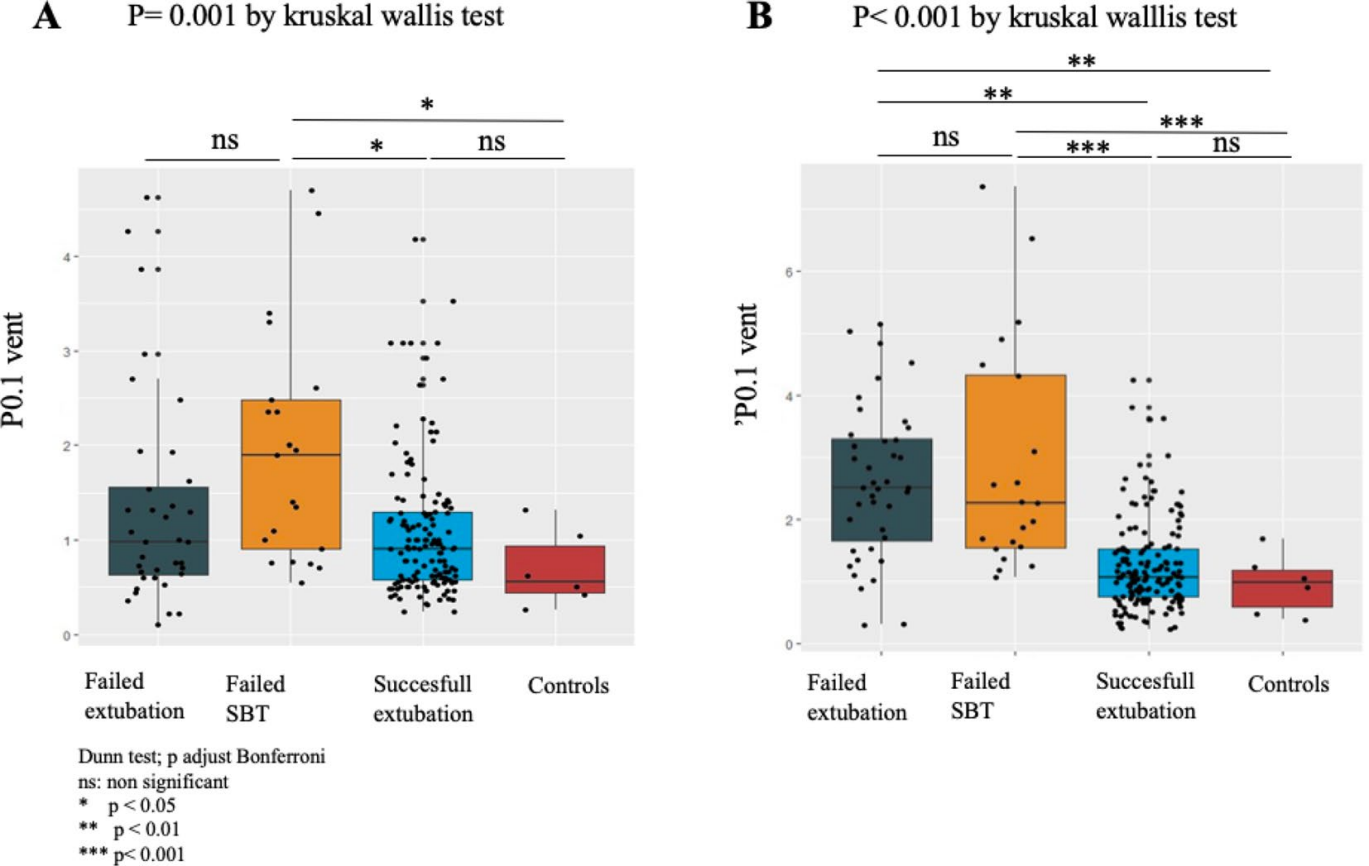
Automated measurements of P_0.1_ (P_0.1__*vent*_) recorded at 0, 15, 30, 45, and 60 min during SBT (PS 0 ZEEP) (**A**) and reventilation period (PS = 8–12 cmH_2_O and PEEP = 5 cmH_2_O) (**B**) in SBT failures, controls, and successful and failed extubations. Box plot: Solid line indicates median, box indicates the interquartile ranges, and whiskers maximum and minimum

The pooled values of P_0.1 vent_ (values recorded at 0, 15, 30, 45 and 60 min) in sessions with different outcomes were analyzed. Outcomes were analyzed on 157 sessions (70%) followed by successful extubations at day 7, 46 sessions (18%) followed by extubation failures at day 7, 21 SBT failures (9%) and 6 sessions (3%) in control group patients (performed in 224 patients). Overall comparison between these 4 groups showed a significant difference in P_0.1_*vent* mean values collected over the 5 time points (0, 15, 30, 45 and 60 min), both during the SBT period (PS0 ZEEP) (*p* = 0. 002) (Fig. 3A) as well as during the reventilation period (*p* < 0.001) (Fig. 3B) with higher P_0.1_*vent* values for SBT failures and extubation failures. The *post-hoc* two by two comparison showed that the pooled values of P_0.1_*vent* collected during SBT and reventilation periods were significantly lower during the SBT period (*p* < 0.001 Wilcoxon test) (supplemental Fig. S1).

In addition, the pooled values of P_0.1 vent_ were compared according to the baseline characteristics of patients. Results for comparisons of mean values of P_0.1_*vent* recorded values during the SBT period and the reventilation period according to the clinical characteristics of the patients and devices used for airway humidification are shown in Table 2.Of note, during the reventilation period, respiratory rate was higher in the extubation failure group than in the success group (24 [19–29] vs. 21 [18–25] breaths per minute respectively, *p* = 0.04) while there was no difference in mean arterial pressure (92 [82–100] vs. 91 [81–100] mmHg, *p* = 0.49) or heart rate (94 [80–111] vs. 91 [80–101] beats per minute, *p* = 0.07).

**Table 2:** Automated measurements of P_0.1_ (P_0.1_*vent*) recorded during SBT (PS 0 ZEEP) and reventilation period (PS = 8–12 cmH_2_O and PEEP = 5 cmH_2_O) and compared according to baseline characteristics

Variable	First period with no pressure support and no PEEP (number of measurements by group) median [IQR]		*P* value
	No	Yes	
Age > 65 years	(124) 0.81 [0.57; 1.31]	(79) 0.97 [0.58 ; 1.39]	0.42
Male gender	(68) 0.72 [0.58 ; 1.28]	(135) 0.98 [0.57; 1.42]	0.16
COPD	(169) 0.82 [0.56 ; 1.30]	(34) 1.20 [0.89 ; 1.42]	**0.04**
OHS	(180) 0.90 [0.58 ; 1.32]	(23) 1.08 [0.59 ; 1.57]	0.29
Obesity	(132) 0.82 [0.56; 1.32]	(71) 0.96 [0.62 ; 1.32]	0.0.36
Cardiac disease	(162) 0.92 [0.58 ; 1.32]	(41) 0.78 [0.56 ; 1.22]	0.75
Neurological disease	(157) 0.91 [0.78 ; 2.06]	(46) 0.86 [0.58 ; 1.33]	0.97
ARDS	(149) 0.80 [0.54 ; 1.28]	(54) 1.08 [0.70 ; 2.02]	**0.003**
Covid19	(185) 0.90 [0.56 ; 1.32]	(18) 0.94 [0.66 ; 1.60]	0.39
Heated humidification	(55) 0.92 [0.60 ; 1.40]	(148) 0.78 [0.50 ; 1.17]	0.07
	**Period of reventilation with pressure support and PEEP = 5 cmH**_**2**_ **O (number of measurements by group) median [IQR]**		**P value**
	No	Yes	
Age > 65 years	(124) 1.08 [0.74 ; 2.00]	(79) 1.32 [0.86 ; 2.09]	0.15
Male gender	(68) 1.12 [0.70 ; 1.59]	(135) 1.22 [0.89 ; 2.18]	0.06
COPD	(169) 1.16 [0.78 ; 1.98]	(34) 1.32 [0.86 ; 2.26]	0.36
OHS	(180) 1.15 [0.76 ; 2.01]	(23) 1.50 [1.01 ; 2.29]	0.15
Obesity	(132) 1.14 [0.77 ; 1.02]	(71) 1.26 [0.85 ; 2.05]	0.68
Cardiac disease	(162) 1.16 [0.79 ; 2.05]	(41) 1.18 [1.88 ; 1.84]	1.78
Neurological disease	(157) 1.22 [0.78 ; 2.06]	(46) 1.12 [0.83 ; 1.77]	0.60
ARDS	(149) 1.10 [0.74 ; 1.68]	(54) 1.51 [1.02 ; 2.56]	**0.005**
Covid19	(185) 1.16 [0.76 ; 2.00]	(18) 1.64 [0.86 ; 2.39]	0.38
Heated humidification	(55) 1.27 [0.91 ; 2.24]	(148) 0.92 [0.57 ; 1.54]	**0.003**

One hundred sixty-nine patients received post-extubation NIV, the median level of pressure support applied was 10 cmH_2_O [8–10] and the median level of PEEP was 5 cmH_2_O [5–8].

### Associations between extubation failure and P0.1 _vent_, hemodynamic and respiratory parameters

Logistic regression analysis revealed a significant association between P_0.1 *vent*_ recorded during the reventilation period (OR = 0.29, 95% CI 0.18–0.43, *p* < 0.001) with extubation success but not for the P_0.1 *vent*_ recorded during the SBT period (OR = 0.74, 95% CI 0.49–1.11, *p* = 0.11). The cross-validated model carried out for P_0.1 *vent*_ recorded during the ventilation period achieved accuracy of 78.7%, and AUC of 0.82 (0.73–0.89) on the test dataset, indicating good predictive performance. As demonstrated in supplemental Fig. S2, there was a moderate and significant correlation between P_0.1 *vent*_ and the ventilator ratio during the SBT and reventilation periods (Spearman’s rho = 0.23 and 0.22 respectively, *p* < 0.001) (refer to supplemental Table S2, which presents extensive results for correlations between P_0.1 *vent*_ and PaO_2_, SpO_2_, respiratory rate and heart rate).

**Fig. 4:**
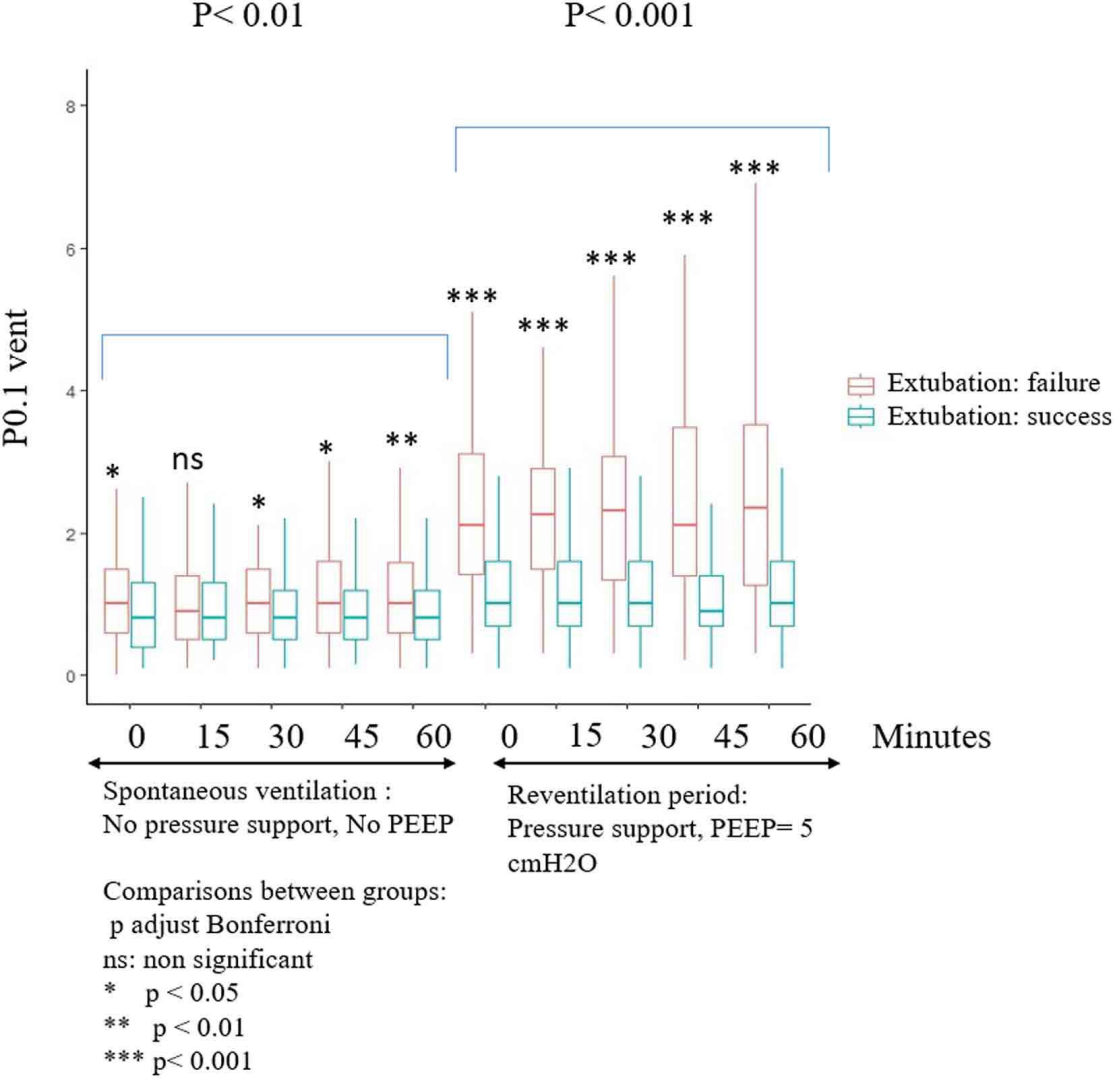
P_0.1 *vent*_ recorded at 0, 15, 30, 45 and 60 min during SBT (PS 0 ZEEP) and reventilation period (PS = 8–12 cmH_2_O and PEEP = 5 cmH_2_O) compared between patients who failed and succeded extubation at day 7

**Fig. 5:**
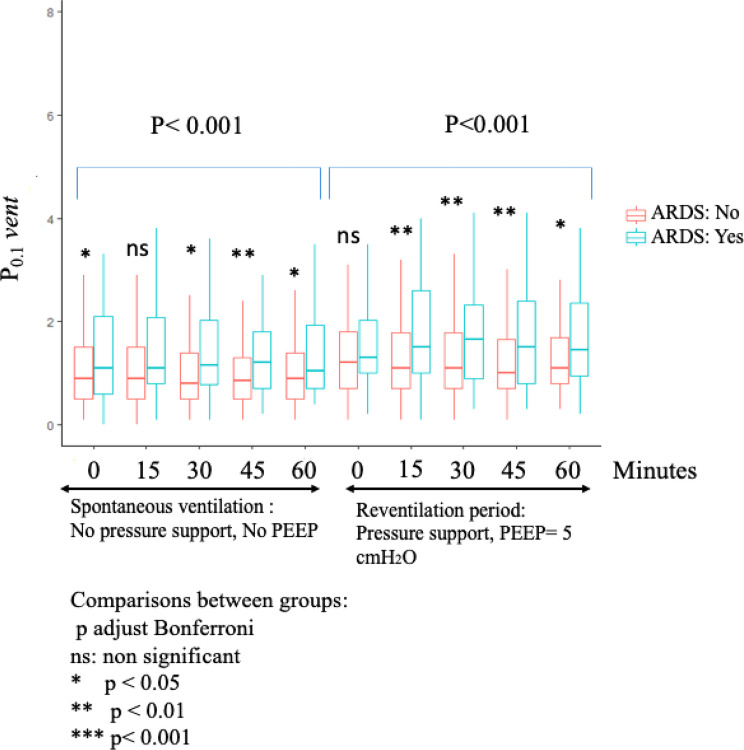
P_0.1_*vent* recorded at 0, 15, 30, 45, and 60 min during SBT (PS 0 ZEEP) and reventilation period (PS = 8–12 cmH_2_O and PEEP = 5 cmH_2_O) compared between patients with and without ARDS. Box plot: Solid line indicates median, box indicates the interquartile ranges, and whiskers maximum and minimum

Repeated values of P_0.1_*vent* were significantly higher in the failure group than in the success group with a considerable overlap of values (Table 3; Fig. 4). In addition, repeated values significantly differed in both the SBT and the reventilation periods whether patients had previous ARDS or not (Table 3; Fig. 5) and according to the device used for humidification of airways (Table 3). On the other hand, repeated P_0.1_*vent* values were not statistically different between COPD and non-COPD patients (*p* = 0.23); nor between obese and non-obese patients (*p* = 0.88).

**Table 3:** Association between baseline characteristics of the patients and repeated values of means of automated measurements of P0.1 (P_0.1 *vent*_)

Model term (repeated values from the start of the first period with no pressure support and no PEEP until the end of the reventilation period with pressure support and PEEP = 5 cmH_2_O) *a*	Estimate	95% CI *b*	*p*-value
Success/ Failure extubation	-0.84	-1.20, -0.51	< 0.001
ARDS Yes/ No	0.40	0.11, 0.73	0.007
Male gender Yes/ No	0.42	0.03, 0.56	0.09
Age > 65 years Yes/No	0.13	-0.15, 0.42	0.38
Obesity Yes/No	-0.03	-0.33, 0.26	0.88
COPD Yes/No	0.22	-0.15, 0.59	0.23
Cardiac Disease Yes/No	0.007	-0.343, 0.36	0.98
Neurological Disease Yes/No	-0.03	-0.36, 0.30	0.85
COVID 19 Yes/No	0.12	-0.37, 0.62	0.61
Heated humidificator Yes/No	0.38	0.06, 0.69	0.015

*a* Adjusted on the level of Pressure Support and the level of Positive End Expiratory Pressure

*b* Coefficient estimates from a linear mixed effects model on the repeated values. A random

intercept was modelled per patient

In addition, the comparison of delta P_0.1_*vent* ((P_0.1_*vent* reventilation - P_0.1_*vent* SBT)/ P_0.1_*vent* SBT) was significantly different according to whether extubation was a success or a failure: delta P_0.1_*vent* extubation successes = 0.21 (0.02–0.62) cmH_2_O vs. P_0.1_*vent* extubation failures = 1.12 (0.54–2.38) *p* < 0.0001.

Analysis of the coefficients of variation over the SBT period and the reventilation period revealed no significant difference between extubation successes (*n* = 157) and failures (*n* = 46). The coefficient of variation for respiratory rate during the SBT was 0.25 for successes vs. 0.29 for failures (*p* = 0.63), during reventilation 0.25 vs. 0.28 (*p* = 0.71). The coefficient of variation for P_0.1_*vent* during the SBT was 0.21 for successes vs. 0.19 for failures (*p* = 0.72), during reventilation 0.6 vs. 0.5 (*p* = 0.42).

## Discussion

We performed a prospective and real-life observational study with exploratory analysis of the values of P_0.1_*vent* recorded in patients tested for their ability to be extubated with success.

First, P_0.1_*vent* values were significantly higher in patients who failed extubation compared to those who succeeded. Additionally, delta P_0.1_*vent* ((P_0.1_*vent* reventilation - P_0.1_*vent* SBT)/ P_0.1_*vent* SBT) was greater in the extubation failure group. Second, P_0.1_*vent* values varied according to the type of airway humidification system used. Furthermore, P_0.1_*vent* values were higher in patients with ARDS compared to non-ARDS patients before extubation. These results suggest that during an SBT, respiratory centers are more activated in patients who fail extubation than in those who succeed.

The accuracy of automatic P_0.1_*vent* estimations provided by modern ICU ventilators has been validated in bench studies, demonstrating high reliability [7, 10, 11, 16]. The key advantages of P_0.1_ and P_0.1_*vent* measurements are that they are non-invasive, imperceptible to patients, and do not influence respiratory centers. Furthermore, because P_0.1_*vent* is estimated at the onset of inspiration—when flow and insufflated volume are zero—it is unaffected by respiratory mechanics. P_0.1_*vent* is also feasible and reliable in the presence of respiratory muscle weakness [12], abnormal respiratory compliance [7], or intrinsic positive end-expiratory pressure.

Another interesting result is the difference in P_0.1_*vent* between the SBT with PS0 and ZEEP and the reventilation period with a pressure support between 8 and 12 cmH_2_O and 5 cmH_2_O of PEEP. The accuracy of P_0.1_*vent* with PS0 is questionable, even though in theory P_0.1_*vent* is calculated at inspiration initiation, when there is no flow variation. We considered two hypotheses. There may have been a technical problem linked to the absence of flow in the ventilator tubes at PS0 ZEEP, which would disrupted the automatic estimations of P_0.1_*vent*. However, bench studies have demonstrated accuracy of the estimation. A physiological explanation could be that there is a ‘recovery’ phase with heightened respiratory center activation immediately following the SBT.

High P_0.1 *vent*_ values were observed not only in patients who failed extubation but also during SBT failures interrupted due to poor tolerance. Conversely, P_0.1_*vent* values were lower in a subset of patients who typically do not require an SBT before extubation. Given that P_0.1_*vent* reliably reflects respiratory center activation [1, 7], these findings suggest variability in respiratory drive activation depending on SBT outcomes. Of course, the large overlap in P_0.1_*vent* values between groups means that this is not clinically useful for prediction of extubation success. However, when used as part of a multimodal approach—including Rapid shallow breathing index (RSBI) and clinical assessment of respiratory mechanics— P_0.1_*vent* could enhance decision-making in weaning and extubation protocols. Moreover, the reventilation period seems to be an interesting period in terms of ventilatory behaviour, which requires further investigation to more accurately categorize patients.

Our results align with previous findings by Esnault et al. [26], who reported heightened respiratory center activity in ARDS patients compared to non-ARDS patients. Notably, our data indicate that elevated respiratory drive persists even after ARDS resolution during mechanical ventilation weaning.

P_0.1_ has also been studied as an indicator of weaning, and high P_0.1_ has reportedly been associated with weaning failure [27–31]. The ventilatory parameters usually analyzed for predicting extubation failure are those measured during the SBT or during screening for mechanical ventilation weaning [19, 30–32]. Few studies have investigated ventilator parameters during the post-SBT reventilation period. Hernandez and al. demonstrated that recovery time to return to baseline minute ventilation could be predictive of extubation failure [33]. Le Marec and al. demonstrated that abnormally high P_0.1_ values may suggest dyspnea and are associated with higher mortality and prolonged duration of MV [34]. Furthermore, in agreement with Le Marec et al., we found that higher P_0.1 *vent*_ values in patients who failed extubation were correlated with the surrogate ventilatory ratio. This suggests an increase in ventilatory dead space in patients who fail extubation.

Wysocki et al. found that the coefficients of variation of inspiratory and expiratory times and tidal volume during an SBT were greater in patients with extubation success [35]. In contrast, our study found no significant differences in P_0.1_*vent* variability between extubation success and failure groups, either during the SBT or the reventilation period.

The strengths of our study are that it is a real-life study, with analysis of a parameter that is easily accessible at the patient’s bedside, requiring neither invasive measurement nor advanced knowledge of mechanical ventilation. To the best of our knowledge, this is the first clinical study to analyze the repeated values of automatic continuous P_0.1_*vent* during the SBT and reventilation periods, with a particular focus on the success or failure of extubation.

The weaknesses of our study are that it is a monocentric study with a limited number of patients. However, to ensure consistency in the data set, a single ventilator was employed for all participants, as evidenced by bench data, which revealed discrepancies between ventilators [8, 9, 15, 16]. We did not compare P_0.1_*vent* with other measurements of respiratory center activation including Neurally adjusted ventilatory assist (NAVA), esophageal pressure, or electrical activity of the diaphragm which were beyond the scope of the study.

## Conclusion

P_0.1_*vent* values are higher in patients who fail extubation, in patients who fail the SBT and in patients who had ARDS. The delta of P_0.1_*vent* values between SBT and reventilation are higher for patients who fail extubation, suggesting higher levels of activation of their respiratory centers. As for previously reported for P_0.1_, the large overlap of values limits the usefulness of P_0.1_*vent* as a reliable tool for predicting extubation failure.

## Electronic supplementary material

Below is the link to the electronic supplementary material.

Supplementary Material 1

## Abbreviations

ARDS: Acute respiratory distress syndrome
BMI: Body mass index
COPD: Chronic obstructive pulmonary disease
ECMO: Extra-corporeal membrane oxygenation
HR: Heart rate
IQR: Interquartile range
MV: Mechanical ventilation
NAVA: Neurally adjusted ventilatory assist
NIV: Non-invasive ventilation
OHS: Obesity hypoventilation syndrome
OTI: Oro-tracheal intubation
PS: Pressure support
PtcCO_2_: Transcutaneous PCO_2_
SAPS: Simplified acute physiology score
SOFA: Sequential organ failure assessment
SBT: Spontaneous breathing trail
RP: Reventilation period
RSBI: Rapid shallow breathing index
RR: Respiratory rate
Vt: Tidal volume
ZEEP: Zero end expiartory pressure
