# Correction: Automatic continuous P_0.1_ measurements during weaning from mechanical ventilation: a clinical study

**DOI:** 10.1186/s13613-025-01497-1

**Published:** 2025-06-17

**Authors:** Flora Delamaire, Maamar Adel, Guillot Pauline, Quelven Quentin, Coirier Valentin, Painvin Benoit, Tadie Jean-Marc, Terzi Nicolas, Gacouin Arnaud

**Affiliations:** 1 Maladies Infectieuses et Réanimation Médicale, CHU de Rennes, Rennes, F-35033 France; 2 Université de Rennes 1, Faculté de Médecine, Rennes, F-35043 France; 3 Inserm-CIC-1414, Faculté de Médecine, Université de Rennes 1, IFR 140, Rennes, F-35033 France

**Correction: Annals of Intensive Care (2025) 15:47**

10.1186/s13613-025-01455-x

In this article the author’s name Flora Delamaire was incorrectly written as Delamaire Flora.

The original article has been corrected.

