# Supplementary material for: Automatic continuous P0.1 measurements during weaning from mechanical ventilation: a clinical study
Source: Ann Intensive Care. 2025 Apr 1;15:47. doi: 10.1186/s13613-025-01455-x (PMC11961779; doi:10.1186/s13613-025-01455-x)
Supplement: Supplementary file 1 — Supplementary Material 1 [file 13613_2025_1455_MOESM1_ESM.docx]

**Supplemental Figure 1**: Pooled values of automated measurements of P_0.1_ (P_0.1_ *_vent_*) recorded at 0, 15, 30, 45 and 60 minutes compared between SBT (PS 0 ZEEP) and reventilation period (PS=8-12 cmH_2_O and PEEP=5 cmH_2_O).


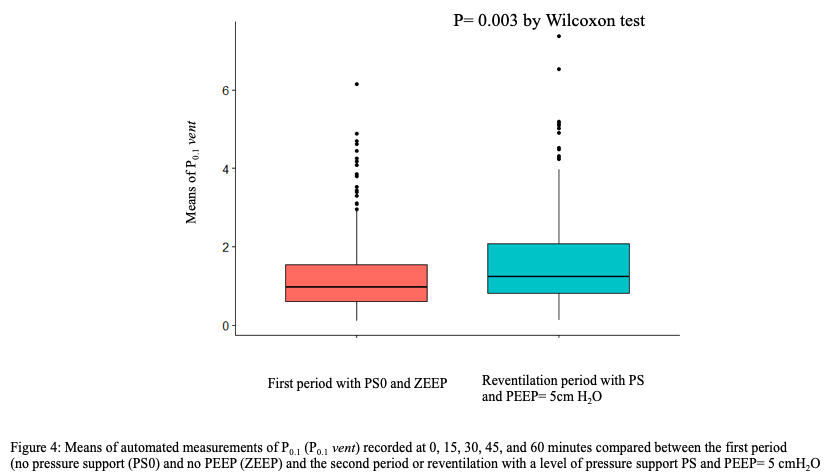


**Supplemental Figure 2:** Area Under the Receiving Operating Characteristic (ROC) curve (AUC) for validation of the model.


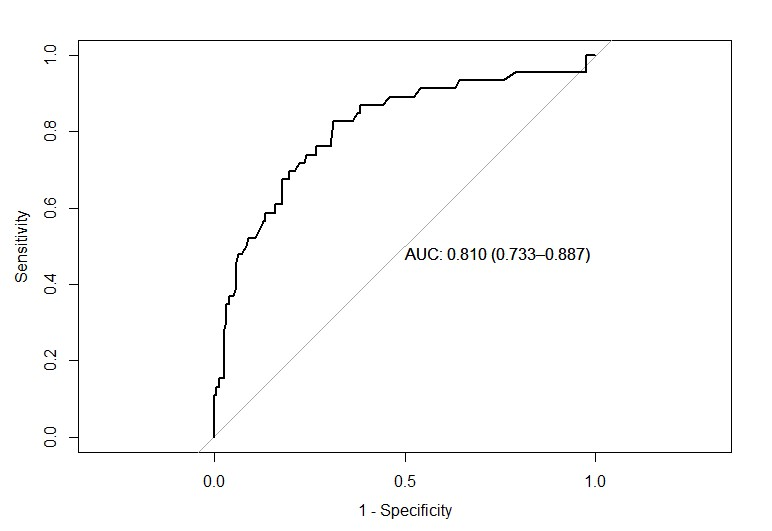


**Supplemental Figure 3**: Correlations between means P_0.1_ *_vent_* measurements recorded at 0, 15, 30, 45, and 60 minutes of SBT (PS 0 ZEEP) and reventilation period (PS=8-12 cmH_2_O and PEEP=5 cmH_2_O) and the ventilator ratio.


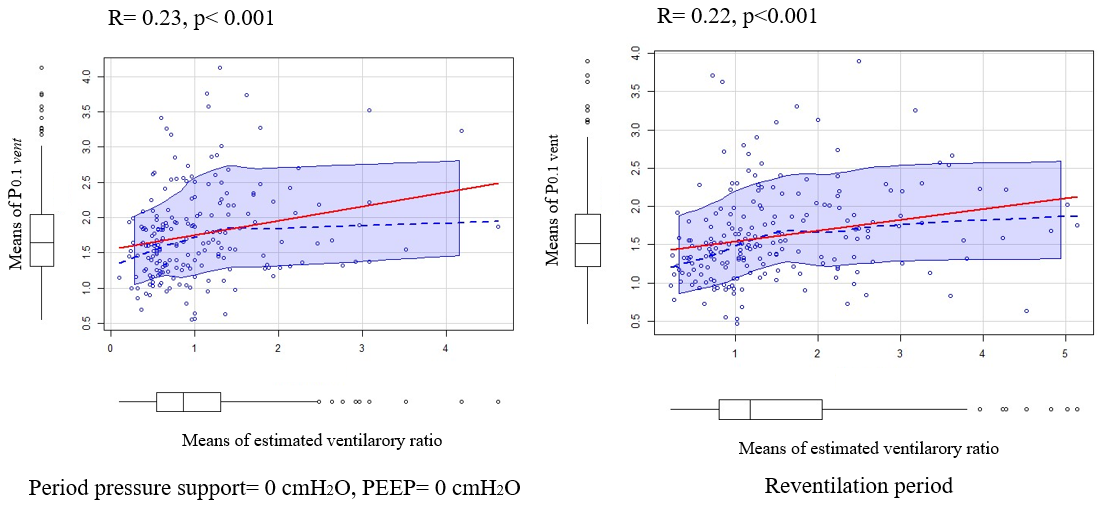


**Supplemental Table 1**: Generally accepted criteria used to identify patients to undergo a SBT

| Resolving the cause of the intubation | |
| --- | --- |
| Cardiovascular criteria | absence of vasopressor or inotrope, hemodynamic stability |
| Neurological criteria | absence of sedation, satisfactory response to simple orders |
| Respiratory criteria | FiO2 ≤ 50%, PEEP < 10 cmH_2_O, effective cough during tracheal aspirations and scanty bronchial secretions |

**Supplemental Table 2:**

Results for correlations between P_0.1_ *_vent_* and PaO_2_, SpO_2_, respiratory rate and heart rate

|  | PS 0 ZEEP | | Reventilation | |
| --- | --- | --- | --- | --- |
|  | Spearman’s rho | P value | Spearman’s rho | P value |
| PaO_2_, mmHg | 0.19 | 0.004 | 0.16 | 0.004 |
| SpO_2_, % | -0.13 | 0.06 | 0.10 | 0.15 |
| Respiratory rate, breah per minute | 0.32 | <0.001 | 0.35 | 0.001 |
| Heart rate, cycles per minute | 0.35 | <0.01 | 0.32 | <0.01 |
